# Single-Pot Synthesis of Biodiesel using Efficient Sulfonated-Derived Tea Waste-Heterogeneous Catalyst

**DOI:** 10.3390/ma12142293

**Published:** 2019-07-18

**Authors:** Umer Rashid, Junaid Ahmad, Mohd Lokman Ibrahim, Jan Nisar, Muhammad Asif Hanif, Thomas Yaw Choong Shean

**Affiliations:** 1 Institute of Advanced Technology, Universiti Putra Malaysia, UPM Serdang 43400, Selangor, Malaysia; 2 Chemical Engineering Department, Khalifa University, Abu Dhabi 127788, United Arab Emirates; 3 School of Chemistry and Environment, Faculty of Applied Sciences, Universiti Teknologi MARA, Shah Alam 40450, Selangor, Malaysia; 4 Center of Nanomaterials Research, Institute of Sciences, Universiti Teknologi MARA, Shah Alam 40450, Selangor, Malaysia; 5 National Centre of Excellence in Physical Chemistry, University of Peshawar, Peshawar 25120, Pakistan; 6 Department of Chemistry, University of Agriculture Faisalabad, Faisalabad 38000, Pakistan; 7 Department of Chemical and Environmental Engineering, Engineering Faculty, Universiti Putra Malaysia, UPM Serdang 43400, Selangor, Malaysia

**Keywords:** waste tea-based catalyst, sulfonation, characterization, PFAD-biodiesel

## Abstract

The main purpose of this manuscript is to report the new usage of tea waste (TW) as a catalyst for efficient conversion of palm fatty acid distillate (PFAD) to biodiesel. In this work, we investigate the potential of tea waste char as a catalyst for biodiesel production before and after sulfonation. The activated sulfonated tea waste char catalyst was characterized using Fourier transform infrared spectroscopy (FTIR), thermogravimetric analysis (TGA), X-ray diffractometry (XRD), elemental composition (CHNS), nitrogen adsorption-desorption using Brunauer-Emmett-Teller (BET) and ammonia-temperature-programmed desorption (NH_3_-TPD). The activated tea waste char catalyst shows higher acid density of 31 μmol g^−1^ as compared to tea waste char of 16 μmol g^−1^ and higher surface area of 122 m^2^/g. The optimum fatty acid conversion conditions were found that 4 wt % of catalyst loading with 9:1 of methanol:PFAD for 90 min of reaction time at 65 °C gives 97% free fatty acid (FFA) conversion. In conclusion, the sulfonated tea waste (STW) catalyst showed an impressive catalytic activity towards the esterification of PFAD at optimum reaction conditions with significant recyclability in five successive cycles without any reactivation step.

## 1. Introduction

Energy is the key factor of the modern world. Fossil fuel is the leading energy provider among all energy basic sources. Depletion of fossil fuel resources is the hot topic among the energy providers and other stakeholders [1]. Biodiesel has been recognized as a substitute to commercial fuel due to similarity in their characteristics. Biodiesel is a blend of long chain alkyl esters produced through transesterification/esterification reaction of oils or fats with alcohol in the presence of catalyst [2]. The ester production is achieved through transesterification using a homogeneous catalyst i.e., potassium and sodium hydroxide [3]. Conventionally hydrochloric and sulfuric acid were used as homogeneous acid catalysts for the esterification process of feedstock containing high free fatty acids (FFA). The disadvantages of homogeneous acid catalysts are saponification or emulsion formation and a complicated and costly separation process is required. Moreover, the purification process is also very complicated and produces a lot of waste [4,5]. 

Biodiesel are mainly prepared from the edible oils obtained from palm, soya, canola and peanut [6] and it is being predicted that 70% of the cost of biodiesel production belongs to the feedstock, which is main hurdle in commercialization of this commodity [7]. To minimize the production cost, a variety of non-conventional (non-edible) oils/fats such as rapeseed, tallow, rubber seed oil, kapok seed oil, jatropha oil, palm fatty acid distillate and used cooking oil were investigated by many researchers as substitute to edible oils [8,9]. Palm fatty acid distillate (PFAD) which is one of the waste by-products is produced during the refining process of palm oil and according to Malaysian Palm Oil Board (MPOB), 782048 tonnes of PFAD was produced in 2018, during the processing of palm oil [10]. PFAD consists of more than 85 wt % of FFA [11]. As the cost of PFAD is much less than refined oil, use of PFAD as feedstock can potentially improve the palm oil waste management and makes more profits for the industry [12]. 

Currently, several research papers have reported on the novel heterogeneous solid catalyst that have just been developed for various applications, however, only a few research papers have reported the utilization of waste materials as a new valuable catalyst for production of biodiesel [13,14]. Heterogeneous catalysts are favored over homogeneous catalysts due to various advantages i.e., they are easily recoverable from the product, less hazardous, compatible in various applications, user friendly, possible in modification, and recyclable which leads to considerable decrease in the production cost [15]. However, the main obstacle faced by researchers and investors is their expensive synthesis which makes the production cost relatively high. In heterogeneous catalysis a support with a high surface area to which the catalyst is affixed is usually very costly and this makes the whole process very expensive [16]. 

Recently, different types of supports material such as zeolites, mixed oxides, sulphated zirconia, and ion-exchange resins have been used for preparation of heterogeneous catalysts for ester production [17,18,19]. However, numerous disadvantages associated with the use of these catalysts were reported in terms of cost, intensive reaction conditions, and poor reusability which ultimately increased the production cost of the biodiesel [20,21]. Alternatively, low cost, inexpensive support material produced from biomass gained attraction in the last few years for heterogeneous catalyst preparation [22,23]. In this connection biomass-based carbon or activated carbon gained attraction because of its diverse physiochemical properties, easy availability, and less costly stuff.

Tea is one of most common drinks around the world. Tea (*Camellia sinensis*) leaves comprises 4–7% cellulose, 5–6% lignin, 5–6% hemicellulose, 14–17% proteins, 3–7% lipids and 5–6% pectin. It is estimated that the production of tea was more than 3.8 million in 2008 [24]. Thousands of tons of tea are used for drinking purposes around the world and this produces a huge pile of solid waste. In Malaysia, tea is used in many different forms and is one of favorite drinks of adults. After preparing tea, the waste is usually disposed of as solid waste. Previously, tea waste was used for different applications such as an adsorbent for industrial wastewater treatment, for preparation of activated carbon (AC) for purification of waste cooking oil esters and for hydrogen production [25,26,27]. However, there is no such study found in literature on tea waste used for the preparation of acidic catalyst and its application for ester conversion. 

PFAD holds a high content of FFA, therefore, the major motive behind this work was to synthesize a novel sulfonated tea waste catalyst for the esterification process of PFAD. Moreover, the detailed characterization and work out of the produced catalyst on the reaction parameters were investigated to achieve better biodiesel production. A reusability study of the produced catalyst has also been studied.

## 2. Materials and Methods 

### 2.1. Materials

The discarded black tea leaves were collected from Mama Restaurant located nearby Universiti Putra Malaysia, Malaysia. PFAD was supplied by Sime Darby Jomalina Sdn Bhd, Malaysia. Methanol (≥99.6%), ethanol (96%), sulfuric acid (96–98%), phosphoric acid (≥98%) of ACS grade was procured from Merck, Malaysia. The fatty acid methyl esters standards were obtained from Sigma Aldrich, Malaysia.

### 2.2. Washing and Cleaning of the Waste Tea Sample

The discarded black tea samples obtained from the local restaurant were subjected to repeated washing with hot water for removal of color, milk, sugar and other impurities. The samples were then boiled until its color had been removed. The obtained samples were then put in oven at 110 °C for 24 h, and later the dried sample was grinded and kept in desiccator for further analysis. 

### 2.3. Chemical Activation of Tea Waste and Char Synthesis

The tea waste (TW) char was synthesized using the chemical activation method. Briefly, 20 g of tea waste was soaked in 10% orthophosphoric acid (H_3_PO_4_) solution for 12 h activation of the tea char waste. After the specified time, it was dried at room temperature and saved for calcination prior to sulfonation. The dried TW powder was carbonized under a stream of N_2_ by raising the temperature to 700 °C for 2 h with a heating rate increase of 5 °C/min. 

### 2.4. Synthesis of Sulfonated Tea Waste (TW) Catalyst

**Scheme 1:**
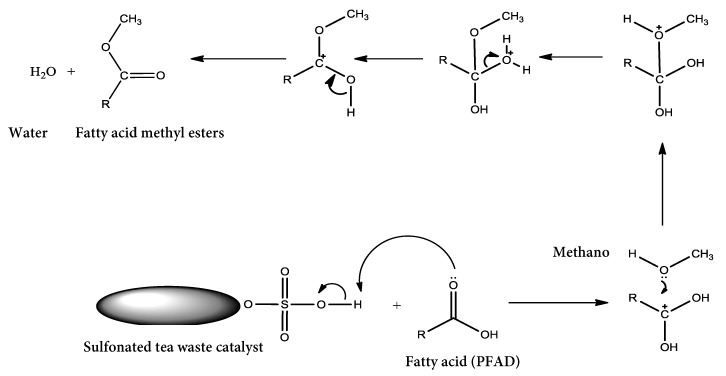
The sulfonated tea waste catalyzed esterification reaction mechanism.

The tea waste and soaked tea waste samples were used to prepare the char samples. Briefly, 10 g of each tea waste and soaked tea waste were heated to produce incomplete carbonized carbon at 400 °C for 5 h in the presence of inert gas at flow of 10 cm^3^ min^−1^. Using hot distilled water, the produced char samples were washed many times until pH was ~7, and then dried in oven at 70 °C for 24 h. From these dried char samples sulfonated acidic catalysts were prepared by mixing 5 g of each char sample with 98% concentrated sulfuric acid in a closed cup autoclave and heated at 160 °C for 6 h. To remove the unreacted sulfuric acid, the obtained catalysts were then washed with hot distilled water (~70 °C). After washing both the catalysts were dried at 70 °C in an oven overnight for complete removal of moisture. The sulfonated tea waste catalyzed esterification reaction mechanism is presented in Scheme 1.

### 2.5. Physical and Chemical Characterization of Sulfonated Tea Waste (STW) Catalyst

The prepared sulfonated tea waste (STW) catalysts were analyzed in detail through several instrumentations. Furthermore, the active sites density was assessed by using the temperature programmed desorption of ammonia (ThermoFinnigan, TPDRO 1100 series, Waltham, MA, USA) which was coupled with thermal conductivity detector. Prior to analysis, the sample was flushed with N_2_ gas for 1 h and heated up to 150 °C for removal of any impurities and water moisture in the sample and reactor system. The sample was further pretreated with ammonia gas for 1 h, at ambient temperature to introduce the ammonia molecules on to the catalyst structure. Again, the sample was flushed with N_2_ gas for 30 min to eliminate any ammonia gas held in the reactor system. The analysis was continued by heating the sample in the temperature range 50 °C to 950 °C at 5 °C min^−1^ in the presence of helium at a flow rate of 20 cm^3^ min^−1^. The amount of ammonia gas desorbed during the heating process was equivalent to the number of active sites on the catalyst. 

The surface characteristics of the STW catalysts were evaluated through N_2_ adsorption/desorption by using BET micromeritics 3 Flex analyzer (Norcross, GA, USA), while, the crystallinity of the catalyst was determined through X-ray diffraction technique, Shimadzu XRD6000 with scan rate at 2° min^−1^ and scan range from 2° to 80°. The morphological analysis of the catalyst was studied via field emission scanning electron microscope (Lausanne, Switzerland). The introduced functional groups after pretreatment and the activation processes were determined by using the Perkin Elmer, 1725X Fourier transform infrared spectrophotometer (Waltham, MA, USA) The sample was scanned from 500 cm^−1^ to 4000 cm^−1^ to get the IR absorption data. Thermal stability of the tea waste char and catalyst were performed using the NETZSCH (STA 449 F3, Netzsch, Selb, Germany) thermogravimetric analyzer. Briefly, a 10 mg of sample was heated from 40 °C to 900 °C at 10 °C min^−1^ in an inert environment at flow rate of 40 mL min^−1^. Elemental composition (carbon, hydrogen, nitrogen and sulfur content) of tea waste char and synthesized catalyst was determined by Vario MRCO cube analyzer by following the EN151004:2005 standard method. 

### 2.6. Catalytic Conversion Study

For esterification of palm fatty acid distillate (PFAD), a 250 mL three-neck round bottom glass flask outfitted with a condenser, thermocouple and magnetic stirrer was used. The reactor was setup in an oil bath (silicon oil) for homogenized heating on a hot plate. Esterification was performed using 2wt % to 5wt % of catalyst loading and varying the methanol to PFAD molar ratio from 3:1 to 12:1. The reaction was allowed to proceed at 30 °C to 70 °C and from 30 min to 120 min. In each run, 25 g of PFAD was used and at the end of each reaction, the sample was separated out by using high-speed centrifuge at 7000 RPM for 10 min. After centrifuge, three layers were obtained: the upper layer was ester, the intermediate was water, and the lower layer was solid catalyst. The catalyst separated was collected for a reusability study. The upper layer (ester phase) was further purified using a rotary evaporator to remove the excess methanol and filtered to remove any solid impurities. The purified esters phase was recovered and preserved for further analysis. 

The FFA conversion was determined based on Equation (1):

(1) $$\mathrm{FFA}\;\mathrm{Conversion}\;\left(\%\right)=\frac{{\mathrm{Av}}_{0}-{\mathrm{Av}}_{t}}{{\mathrm{Av}}_{0}}\;\times 100$$

where A_Vo_ is the initial acid value and A_Vt_ is the acid value measured at time t.

### 2.7. Reusability of The STW Catalyst

The reusability ability of the STW catalyst was investigated. After the reaction, the separated catalyst was collected and washed with acetone and hexane for removal of the trapped solvent and then the washed catalyst was dried at 70 °C for 6 h. After every cycle, acid content and ester conversion were determined to check the efficiency of the catalyst. 

### 2.8. Statistical Analysis

Analysis of three different samples was carried out and results were reported as mean ±SD and presented in the form of bar graph in figures. Each sample was prepared and measured separately in triplicate for all the reported data. 

## 3. Results and Discussion

### 3.1. Acid Density Analysis

Surface area and active acid sites density are the two main factors to determine the level of catalyst activity. Therefore, ammonia temperature programmed desorption (NH_3_-TPD) was used for measuring the acid strength of tea waste char and sulfonated tea waste catalysts. Table 1 depicts that the sulfonated tea waste (STW) catalyst pre-treated with H_3_PO_4_ exhibited the highest acid density of 31 μmol g^−1^ as compared to the tea waste (TW) char, which only went through the soaking process with H_3_PO_4_, and showed less total acid density of 16.8 μmol g^−1^. It was also observed that the treatment of the incomplete carbonized tea waste with the phosphoric acid increased surface area of the TW char itself, which gave extra anchor sites for the attachment of the active sulfonic groups on the catalyst structure.

### 3.2. Surface Analysis

In order to get detailed surface characteristics of the catalyst Brunauer-Emmet-Teller (BET) surface area analysis was carried out and the surface area, pore volume and pore size of tea waste char and tea waste char sulfonated catalysts thus determined are presented in Table 1. It was observed that after sulfonation tea waste char surface area was increased from 50 m^2^/g to 122 m^2^/g, and this was indicative of the fact that chemical activation had opened the surface pores of the char surface.

### 3.3. Functional Group Analysis 

**Figure 1:**
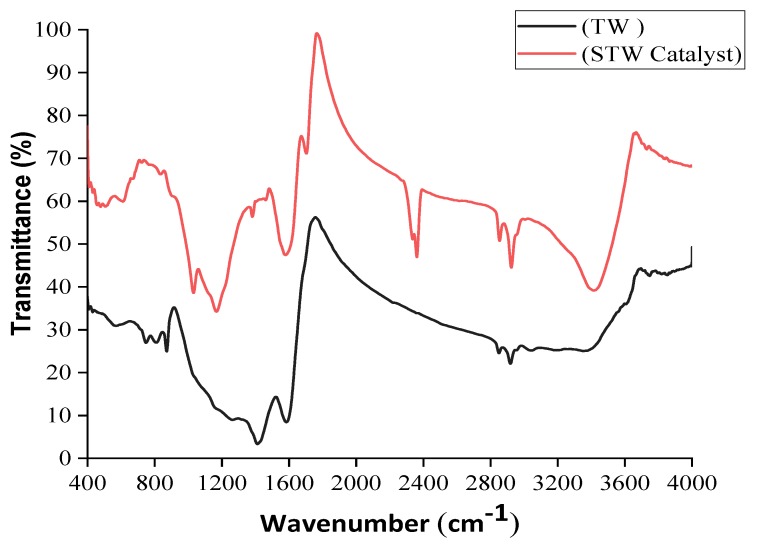
FT-IR spectra of tea waste (TW) char and sulfonated tea waste (STW) catalyst.

Figure 1 reveals the IR spectra of the tea char and tea char based activated catalyst. The presence of sulfonic groups (−SO_3_H) can be detected by the IR absorption at 1190 cm^−1^ for −SO_2_ asymmetric stretching and at about 1040 cm^−1^ for the −SO_2_ symmetric stretching mode [28,29]. Almost similar results were also reported by Lokman et al. [30] for a sulfonated carbon catalyst obtained from waste carbohydrates. This shows that the sulfonation process introduced or generated the sulfonic functional groups that covalently bonded on the carbon chain structure of the catalyst. Even though, the hydroxyl group (−OH) was clearly observed by the appearance of the broad absorption peak at around 3500 cm^−1^ to 3000 cm^−1^. Another peak was noticed at 1590 cm^−1^ corresponding to the carbonyl, C=O stretching mode for the carboxylic groups, −COOH.

### 3.4. Morphological Analysis 

**Figure 2:**
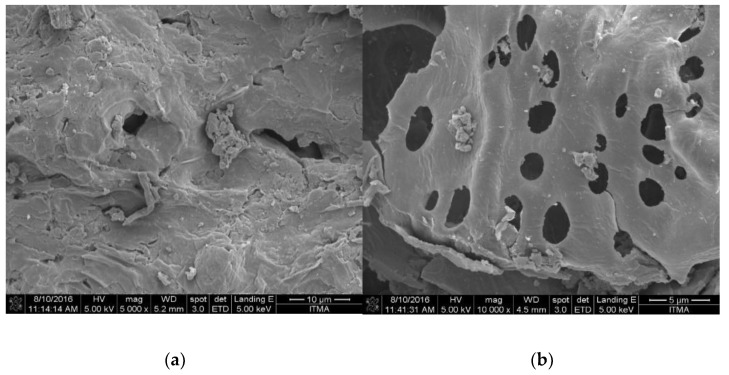
Field emission scanning electron microscopy images (FESEM) images of (**a**) TW-Char (5000×); (**b**) STW catalyst (10,000×).

The field emission scanning electron microscopy images (FESEM) of tea waste char and sulfonated tea waste catalyst are illustrated in Figure 2. It was found that the FESEM image of sulfonated tea waste char was rough, amorphous and had a heterogeneous structure, whereas the sulfonated tea waste char catalyst which was initially treated with phosphoric acid showed a very clear porous structure on the char surface. Konwar et al. [31] observed that the pretreatment of carbon catalyst with phosphoric acid has led to a porous carbon structure with high surface area associated with highly active sites and these findings agree well with our results.

### 3.5. Elemental Composition

Elemental composition of tea waste char and sulfonated catalyst is presented in Table 2. The results indicate that sulfur contents have increased after sulfonation with successful dispersion on the char surface. 

### 3.6. XRD Analysis

**Figure 3:**
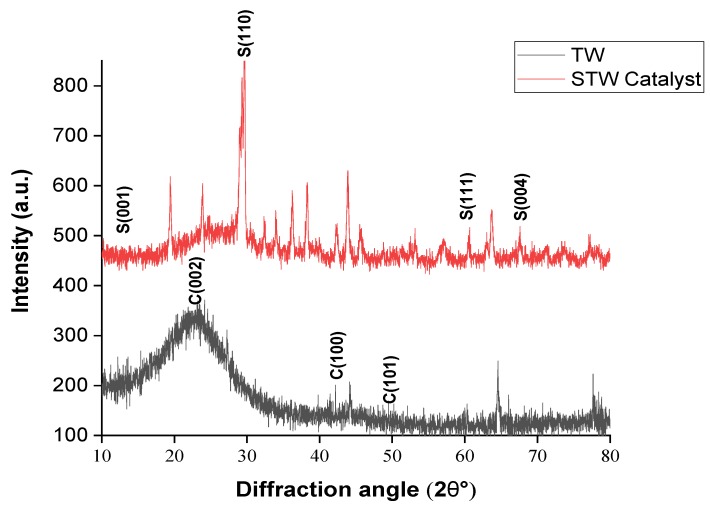
XRD diffractogram of sulfonated-derived tea waste; tea char (black) and sulfonated tea waste acidic catalyst (red).

XRD patterns of tea waste char and sulfonated tea waste char catalyst is presented in Figure 3. It was observed that both tea waste char and sulfonated tea waste (STW) catalyst exhibit identical trends of XRD peaks, broad and weak C(002), C(100) and C(101) around 2θ from 25°–28°, and 40°–50°, respectively. Whereas, STW catalyst showed the other peaks at around 12°, 30°, 56°–68° belong to S(001), S(110), S(111) and S(004) respectively [32]. The presence of sulfur was identified also during the Energy-dispersive X-ray spectroscopy (EDX) analysis (Table 2). The peaks in this region correspond to the amorphous carbon structure which is in accord with the results reported by Yu et al. [33] and Konwar et al. [31].

### 3.7. Thermal Stability Analysis

**Figure 4:**
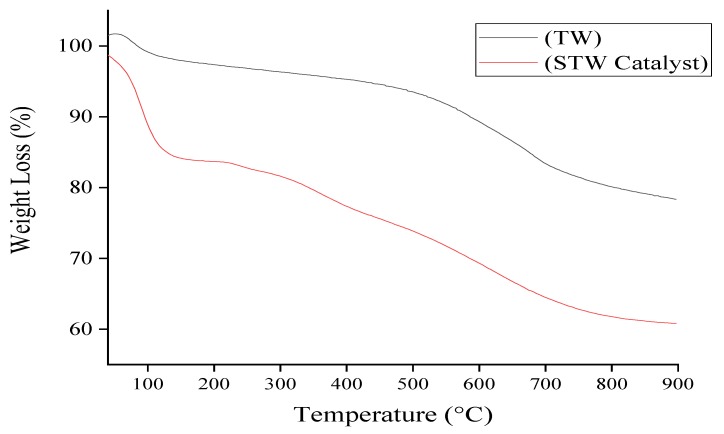
Thermograms of tea waste (TW) char and sulfonated tea waste (STW) acidic catalyst.

The thermal stability of tea waste char and tea waste char based acidic catalysts were examined by thermogravimetric analysis. Figure 4 illustrates the mass loss trend of char and catalyst with increasing temperature under inert atmosphere. The tea waste char sample showed a linear kind of trend and by increasing the temperature from 40 °C to 400 °C no appreciable mass loss was observed, from 400 °C to 800 °C maximum loss in mass was noted and then onward no mass loss was detected. Whereas STW showed a different trend, with maximum mass loss in between 40 °C to 250 °C which can be collectively contributed to the evolution of moisture and decomposition/evaporation of S contents present in −SO_3_H. Further mass loss could be credited to the incomplete pyrolysis of tea waste because initially tea waste pyrolysis was performed at 400 °C. Thermogravimetric analysis (TGA) results showed that the tea waste char catalysts were thermally stable for the sulfonation process. 

### 3.8. Factors Affecting Esterification Conversion

In this study, a sulfonated tea waste (STW) catalyst was used to optimize the four-reaction parameters i.e., methanol:PFAD, catalyst loading, reaction temperature and time for the conversion of PFAD to biodiesel. Efforts were done to utilize the tea waste char as a catalyst, but it gave ~20% FFA conversion of PFAD. 

#### 3.8.1. Effect of Methanol:PFAD Molar Ratio on FFA Conversion

**Figure 5:**
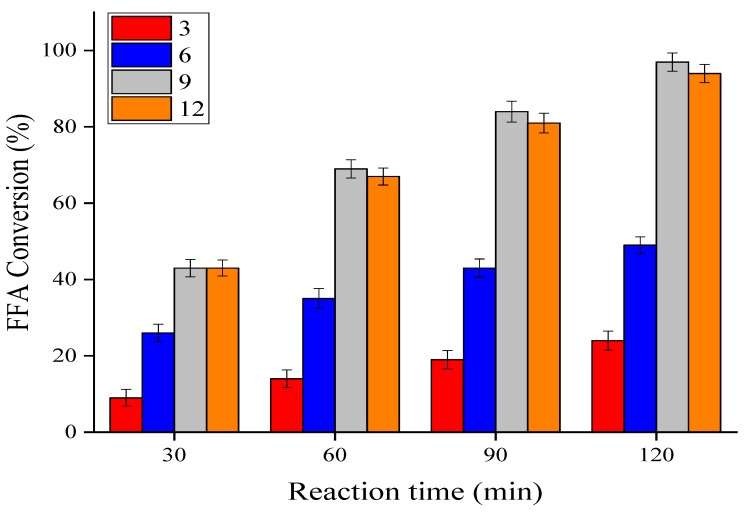
Effect of methanol to palm fatty acid distillate (PFAD) ratio on free fatty acids (FFA) conversion.

Molar ratio is one of the most important factors in the esterification reaction and generally conversion of fatty acid to esters needs three moles of alcohol for one mole of oil. Nevertheless, esterification is a reversible reaction and to move the equilibrium to the forward direction extra amounts of alcohol are required. In this study, several molar ratios (3:1 to 12:1) were tried to achieve maximum conversion of FFA (Figure 5). In this case, 9:1 methanol to PFAD molar ratio was found to be the best ratio for maximum FFA conversion of PFAD of 97%. It was also noted that by enhancing the molar ratio from 9:1 to 12:1, a negative impact was observed and FFA conversion decreased from 97% to 89%. In an earlier study Mardhiah et al. [4] observed highest FFA conversion at 12:1 and a negative impact on additional enhancement in molar ratio of methanol to oil. This negative impact produced by an increase in molar ratio of methanol to oil may be attributed to the deactivation of the catalyst due to hindering of the active groups on the surface of catalyst which ultimately shifted the equilibrium in the reverse direction. These observations are also in agreement with reported literature [34,35]. 

#### 3.8.2. Effect of Catalyst Loading

**Figure 6:**
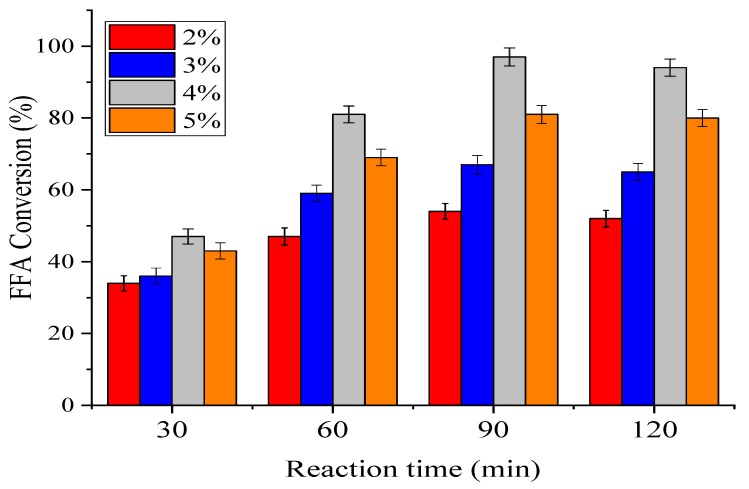
Effect of catalyst loading on FFA conversion.

The catalyst helps to increase the reaction rate by decreasing the activation energy of the process, and therefore, increasing the amount of catalyst surely speeds up the conversion of reactants into products [36]. In this study, different catalyst concentration ranges from 2 wt % to 5 wt % was used for FFA conversion. It was noted that by raising the amount of catalyst the FFA conversion was also increased as indicated in Figure 6. The peak FFA conversion was achieved at 4 wt % of catalyst, however, further increase in the catalyst amount showed negative effect and a reduction in FFA conversion was noted. It may probably be credited to the difference of mass transfer in between FFA content and catalyst active sites and therefore, excess amount of catalyst disturbed the reaction equilibrium [37]. In earlier reported studies a similar trend was also observed, and the authors noted that, after a certain limit further increase in catalyst showed negative results [38,39].

#### 3.8.3. Effect of Reaction Temperature 

**Figure 7:**
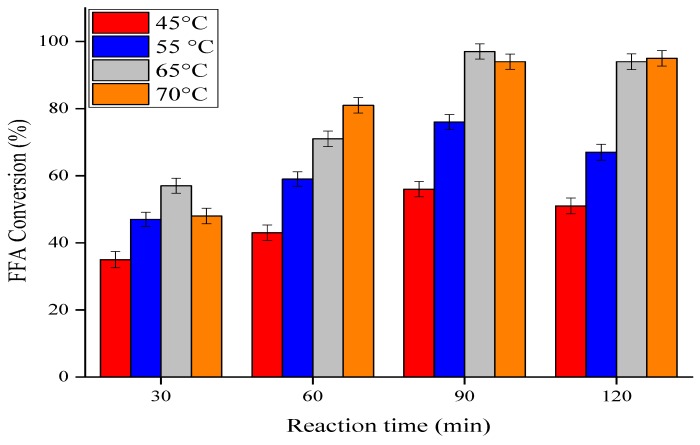
Effect of reaction temperature on FFA conversion.

Temperature directly effects any reaction rate and it is also a cost dependent variable of any process. In order to confirm this, different temperatures range from 45 °C to 70 °C were used for the esterification of PFAD, keeping other parameters constant i.e., catalyst loading 4 wt %, methanol:PFAD molar ratio 9:1 and reaction time 90 min at 350 RPM (Figure 7). It was observed that by rising temperature from 45 °C to 65 °C the FFA conversion improved from 35% to 97%. Temperatures above 65 °C to 75 °C showed negative impact and the end result was a decrease in FFA conversion from 97% to 94%. There are many reasons behind this decrease in FFA conversion after a certain limit. However, the most important one is the evaporation and bubbling out of methanol at a temperature higher than the boiling point of methanol which results in a continuous decrease in methanol content in the reaction mixture and thus sufficient methanol is not available for FFA conversion at temperatures more than the boiling point of methanol [40,41].

#### 3.8.4. Effect of Reaction Time on FFA Conversion

**Figure 8:**
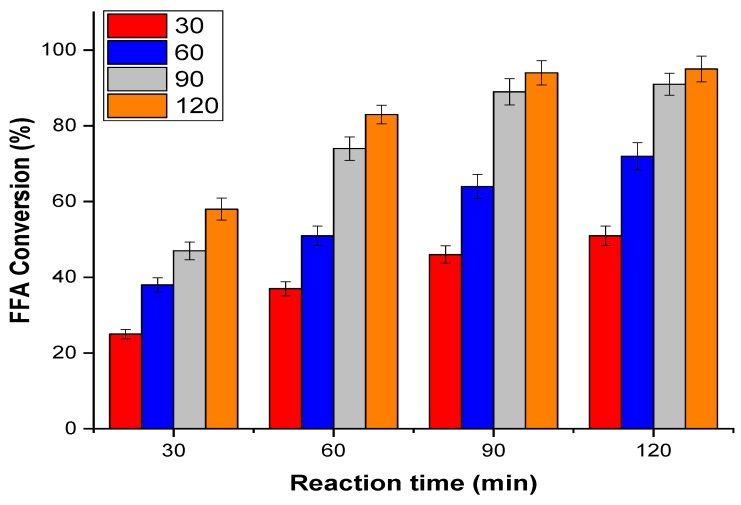
Effect of reaction time on FFA conversion.

The influence of reaction time was investigated from 30 min to 120 min keeping other parameters constant such as catalyst loading of 4 wt %, methanol to PFAD molar ratio 9:1 and reaction temperature 65 °C at 350 RPM (Figure 8). It was observed that with an increase in reaction time FFA conversion was observed to increase. Maximum FFA conversion of 97% was achieved at 90 min of reaction time; however, further increase in reaction time reduced the FFA conversion. These observations are in conformity with earlier reported results [42,43]. There are several possible reasons of a decrease in the FFA conversion after 90 min, such as increase in water concentration within the reaction media which has a profound effect on the catalyst activity and ultimately FFA conversion is decreased. Moreover, long reaction time also weakens the bonding of the active group from the carbon surface and thus an increase in leaching of sulfonic groups adversely affects the catalyst activity [41].

### 3.9. Regeneration and Reusability of STW Catalyst

**Figure 9:**
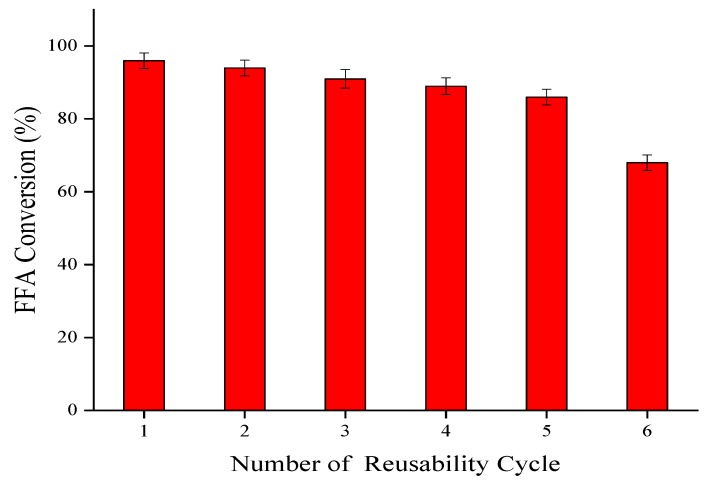
Regeneration/reusability of the STW catalyst.

The regeneration and reusability of STW was evaluated and the obtained results are illustrated in Figure 9. The esterification reaction was performed at catalyst loading of 4 wt %, methanol to PFAD ratio as 9:1, reaction temperature 65 °C and reaction time 90 min. At the end of each run, the catalyst was recovered by filtration and washed with acetone followed by ethanol and distilled to purify it from all the trapped/adsorbed unwanted polar and non-polar compounds. It was observed that the STW catalyst could be used up to five consecutive runs without losing much activity. STW showed a FFA conversion of the PFAD from 96% to 68% after being reused for six times. This indicates that the STW catalyst works well until five cycles. The regeneration and reusability of STW catalyst showed that it has the potential to be used on a commercial scale. 

### 3.10. Comparison of Catalytic Activity of Different Residues Valorized as Sulfonated Char Catalysts for Esterification

A summary of the catalytic activity of different residues valorized as catalyst support in the synthesis of sulfonated char catalysts to produce esters, or biodiesel is presented in Table 3. Sulfonated sugarcane bagasse, sulfonated multiwalled carbon nanotubes and sulfonated magnetic solid acid catalysts need higher methanol to PFAD molar ratio and reaction temperature, whereas, sulfonated sugarcane bagasse requires more catalyst loading as compared to the sulfonated tea waste catalyst. However, sulfonated tea waste (produced in this study) showed better reaction time as compared to previously synthesized catalysts. The catalyst produced in this study showed better reusability as compared to sulfonated sugarcane bagasse, cacao shell-derived solid acid catalyst, sulfonating carbonized corn straw and sulfonated magnetic solid acid catalysts (Table 3). Hence, the catalyst produced from sulfonated tea waste in the present study is expected to be an effective solid acid catalyst for the esterification of PFAD and could also be used for other high FFA feedstocks.

## 4. Conclusions

In short, a highly active sulfonated tea waste (STW) catalyst was prepared from the waste material by wet impregnation method using sulfuric acid as an active agent for PFAD conversion through esterification. The catalytic activity was studied to investigate the catalyst efficiency by studying the process optimization conditions. The optimization conditions revealed that the catalyst worked very well at a low temperature of 65 °C, with catalyst loading of 4 wt %, by using 9:1 PFAD to methanol ratio for 90 min of reaction time and gave 97% FFA conversion. The STW catalyst characterization results revealed that it showed very high acid density, external thermal stability and a porous nature. Regeneration and reusability of STW catalyst shows very good results up to five cycles with good FFA conversion ability. This indicates that catalyst is feasible on a commercial scale because of its diverse properties i.e., it is inexpensive, works on lower temperature and can be used multiple times. The authors believe that the STW based solid acid catalyst is an appealing catalyst for PFAD conversion into esters. 

## Figures and Tables

**Table 1:** Acid density and surface characterization of tea waste char and sulfonated tea waste catalyst.

Sample	NH_3_-TPD Analysis	Surface Characteristics		
	Acid Sites Density (μmol g^−1^)	Average Surface Area (m^2^ g^−1^)	Average Pore Volume (cm^3^ g^−1^)	Average Pore Size (nm)
Tea waste char	16.8 ± 0.48	59 ± 0.98	0.02 ± 0.002	4.8 ± 0.20
Sulfonated tea waste catalyst	31 ± 1.21	122 ± 1.60	0.11 ± 0.015	3.6 ± 0.31

**Table 2:** Elemental composition of tea waste (TW) char and synthesized sulfonated tea waste (STW) catalyst.

Samples	C	H	N	S	O*
Tea waste char	64.65 ± 2.15	3.94 ± 0.10	2.69 ± 0.11	0.28 ± 0.12	28.19 ± 1.25
Sulfonated tea waste catalyst	58.55 ± 1.68	0.77 ± 0.07	2.71 ± 0.09	2.67 ± 0.10	35.3 ± 0.90

* Calculated by difference.

**Table 3:** Catalytic activity of different residues valorized as sulfonated char catalysts for esterification.

Catalysts	Feedstock	Reaction Parameters				Reusability	References
		Methanol/Oil or PFAD Ratio	Catalyst Loading (%)	Temperature (°C)	Time (h)		
Sulfonated sugarcane bagasse	PFAD	20:1	11.5	170	0.5	1 run	[44]
Sulfonated multiwalled carbon nanotubes	PFAD	20:1	3	170	2	5 runs	[45]
Sulfonated magnetic solid acid catalysts	Oleic acid	12:1	9	90	5	4 runs	[46]
Cacao shell-derived solid acid catalyst	Oleic acid	7:1	5	42	4	4 runs	[47]
Sulfonating carbonized corn straw	Oleic acid	7:1	7	60	4	-	[48]
Sulfonated tea waste	PFAD	9:1	4	65	1.5	5 runs	This study
